# Urinary Bisphenol A Levels in Young Men: Association with Reproductive Hormones and Semen Quality

**DOI:** 10.1289/ehp.1307309

**Published:** 2014-05-01

**Authors:** Tina Harmer Lassen, Hanne Frederiksen, Tina Kold Jensen, Jørgen Holm Petersen, Ulla N. Joensen, Katharina M. Main, Niels E. Skakkebaek, Anders Juul, Niels Jørgensen, Anna-Maria Andersson

**Affiliations:** 1 University Department of Growth and Reproduction, Rigshospitalet, Copenhagen, Denmark; 2 Department of Environmental Medicine, Institute of Public Health, University of Southern Denmark, Odense, Denmark; 3 Department of Biostatistics, Faculty of Health and Medical Sciences, University of Copenhagen, Denmark

## Abstract

Background: Few human studies have examined bisphenol A (BPA) exposure in relation to semen quality and reproductive hormones in men, and results are divergent.

Objectives: We examined associations between urinary BPA concentration and reproductive hormones, as well as semen quality, in young men from the general population.

Methods: Our study population consisted of 308 young men from the general population. Urinary BPA concentration was measured by isotope dilution TurboFlow-liquid chromatography–tandem mass spectrometry. We used multiple linear regression analysis to estimate associations between BPA concentration and reproductive hormones and semen quality, adjusting for confounding factors.

Results: We found that 98% of the men had detectable urinary levels of BPA. Median (5th–95th percentiles) BPA concentration was 3.25 ng/mL (0.59–14.89 ng/mL). Men with BPA concentrations above the lowest quartile had higher concentrations of serum testosterone, luteinizing hormone (LH), estradiol, and free testosterone compared with the lowest quartile (*p*_trend_ ≤ 0.02). Men in the highest quartile of BPA excretion had on average 18% higher total testosterone (95% CI: 8, 28%), 22% higher LH (95% CI: 6, 39%), and 13% higher estradiol (95% CI: 4, 24%) compared with lowest quartile. Men in the highest quartile of BPA also had significantly lower percentage progressive motile spermatozoa compared with men in the lowest quartile (–6.7 percentage points, 95% CI: –11.76, –1.63). BPA was not associated with other semen parameters. Adjusting for dietary patterns did not influence the results.

Conclusions: The pattern of associations between BPA and reproductive hormones could indicate an antiandrogenic or antiestrogenic effect, or both, of BPA on the hypothalamic–pituitary–gonadal hormone feedback system, possibly through a competitive inhibition at the receptor level. However, additional research is needed to confirm our findings and to further test the suggested potential mechanisms.

Citation: Lassen TH, Frederiksen H, Jensen TK, Petersen JH, Joensen UN, Main KM, Skakkebaek NE, Juul A, Jørgensen N, Andersson AM. 2014. Urinary bisphenol A levels in young men: association with reproductive hormones and semen quality. Environ Health Perspect 122:478–484; http://dx.doi.org/10.1289/ehp.1307309

## Introduction

Concern has been raised about the endocrine-disrupting effects of exposure to the widely used chemical bisphenol A (BPA) including its effects on male reproductive health (Richter et al. 2007; vom Saal et al. 2007). BPA is used to manufacture polycarbonate plastics and epoxy resins and is present in a wide variety of consumer products. Human exposure to BPA is believed to occur mainly through the diet due to leaching of BPA from packaging materials into food items. Other possible routes of exposure are inhalation of indoor dust and dermal contact (Vandenberg et al. 2013). After uptake, BPA is almost exclusively excreted in the urine, with an elimination rate of hours (Völkel et al. 2002, 2005). Widespread and continuous exposure to BPA in humans has been confirmed by biomonitoring studies from general populations, in which > 80% of participants had detectable levels of BPA in urine (Calafat et al. 2008; Kasper-Sonnenberg et al. 2012).

Since the 1930s, BPA has been known to possess estrogenic activity (Dodds and Lawson 1936). *In vitro* studies have demonstrated that BPA can interact with both estrogen receptor subtypes (ERα and ERβ), leading to estrogenic effects, or it can act in competition with 17β-estradiol to exert antiestrogenic effects (Gould et al. 1998; Kuiper et al. 1997; Li et al. 2012). In addition, BPA has also been shown *in vitro* to act as an androgen receptor (AR) antagonist (Bonefeld-Jørgensen et al. 2007; Kitamura et al. 2005).

Rodent studies have demonstrated adverse effects on markers of male reproductive health due to BPA exposure early in life or in adulthood (Dobrzynska and Radzikowska 2013; Qiu et al. 2013; Quignot et al. 2012; Richter et al. 2007; vom Saal et al. 1998), although not all rodent studies have shown the same effects (e.g., Ashby et al. 2003; Ema et al. 2001). In humans, few studies have examined BPA exposure in association with reproductive hormones or semen quality, and the results are divergent. This may be due in part to differences in study population selections of, for example, occupationally exposed men (Hanaoka et al. 2002; Li et al. 2011), fertile men (Mendiola et al. 2010), or men attending fertility clinics (Meeker et al. 2010a, 2010b). To our knowledge, only two studies have previously examined associations between urinary BPA excretion and reproductive hormones in men from the general population (Galloway et al. 2010; Kim et al. 2013).

The aim of the present study was to examine the relationship between the urinary concentration of BPA and reproductive hormones and semen quality in a group of 308 healthy, young men from the general population.

## Materials and Methods

*Study population*. The study population consisted of young Danish men from the general population who participated in a large on-going prospective study of reproductive health for which approximately 300 new men have been recruited each year since 1996, as previously described (Jørgensen et al. 2002, 2012). Recruitment for the onging study takes place when the men attend a compulsory physical examination for military service that all Danish men, except those suffering from severe chronic disease, are required to undergo. The men included in the present study were recruited from September 2008 through June 2009. In brief, the participants provided a spot urine sample and a semen sample, had a nonfasting blood sample drawn, underwent a physical examination, and handed in a completed questionnaire, generally all within approximately 1 hr. Self-reported time since previous ejaculation was recorded. Height and weight of the men were measured at the physical examination. All semen, urine, and blood samples were collected between 0840 and 1230 hours. The questionnaire contained information on demographics, health and lifestyle factors (including alcohol intake and smoking), and information on dietary intake from a validated 136-item food-frequency questionnaire reflecting dietary intake for the 3 months before participation in the study (for further details, see Jensen et al. 2013). Responses from the food-frequency questionnaire have previously been used to calculate percentage intake of total dietary fat, saturated fat, protein, and carbohydrate (Jensen et al. 2013). More detailed information on the BPA distribution of the participating men can be found in Frederiksen et al. (2014). We also had information on urinary excretion of phthalate metabolites from an earlier study on the same men, as described in detail by Joensen et al. (2012).

Of the initial study group of 313 men, 5 were excluded: 1 because of abuse of anabolic steroids, 2 because of missing blood samples, and 2 because of missing questionnaire information, leaving 308 men eligible for analyses. In addition, we excluded two men from the semen analyses because of obstructive azoospermia. Because of missing data on covariates or outcomes, the analyses with reproductive hormones were based on 303 men, and semen analyses on 298 men.

Ethical approval was attained from the local ethical committee of the Danish National Committee on Health Research Ethics, Copenhagen, Denmark (H-KF-289428), and informed written consent was obtained from all participants before participation.

*Chemical analyses*. Following a preceding enzymatic deconjugation of the samples, we analyzed the urinary content of total BPA (the sum of unconjugated and enzymatically deconjugated) by a newly developed isotope dilution TurboFlow liquid chromatography–tandem mass spectrometry method for quantitative determination. We used a Thermo Scientific Aria TLX-2 LC equipped with TurboFlow Cyclone P columns (0.5 mm × 50 mm) and Hypersil Gold aQ columns (4 mm × 50 mm, 3-μm particle size) (both from Thermo Scientific, Franklin, MA, USA) coupled to TSQ Vantage triple quadrupole mass spectrometer (Thermo Fisher Scientific, San Jose, CA, USA), in combination with Aria operating software 1.6.2 and Xcalibur 2.1.0.1139 system software (ThermoFinnigan, Bellefonte, PA, USA). We have previously published details of the method in Frederiksen et al. (2013). In short, samples were analyzed in eight batches during a period of 2 weeks. Each batch included standards for calibration curves, about 40 unknown samples, 2 blanks, 2 urine pool controls, and 2 urine pool controls spiked with BPA standards at low or high level, respectively. The analytical interday variation, expressed as the relative standard deviation (RSD), was ≤ 10% for most analytes and the recovery of spiked samples was > 99%. The level of detection (LOD) for BPA was 0.12 ng/mL.

Urinary osmolality, which reflects urinary dilution, was measured by the freezing point depression method using an automatic cryoscopic osmometer (Osmomat® 030; Gonotec, Berlin, Germany). For each nine sample measurements, a standard urine pool was measured. Mean urinary osmolality for this standard pool (*n* = 36) was 0.820 Osm/kg (osmoles per kilogram) with an RSD of 1.65%. The median (range) osmolality of the urine samples included in the present study was 0.860 Osm/kg (0.110–1.477 Osm/kg).

*Semen analysis*. We performed the semen analysis as previously described (Jørgensen et al. 2012). Briefly, semen volume was assessed by weight, with the assumption that 1 g semen has a volume of 1 mL. Sperm concentration was assessed using a Bürker-Türk hemocytometer (Paul Marienfeld GmbH & Co. KG, Lauda-Königshofen, Germany), and only sperm with tails were counted. Total sperm count was calculated (semen volume × sperm concentration). Sperm motility was assessed by classifying the sperm according to World Health Organization (WHO) criteria as either progressive motile (class A + B), locally motile (class C), or immotile (class D) (WHO 1999). For sperm morphology evaluation smears were prepared, Papanicolaou stained, and assessed according to strict criteria as described by WHO (1999).

*Reproductive hormone analyses*. Participants had nonfasting blood samples drawn from the cubital vein, and serum was stored at –20°C until analyses. The reproductive hormones were analyzed during June–August 2010. Serum concentrations of follicle-stimulating hormone (FSH), luteinizing hormone (LH), and sex hormone–binding globulin (SHBG) were measured by time-resolved immunofluorometric assays (Delfia immunodiagnostics system; PerkinElmer, Wallac, Turku, Finland). The intra- and interassay coefficients of variation (CVs) were < 6% for these three assays. Testosterone (total testosterone, T) and estradiol (E_2_) were measured by fluoroimmunoassay (Delfia) with CVs for both assays of ≤ 8%. Inhibin B was determined by a double antibody enzyme–immunometric assay (Beckman Coulter Inc., Brea, CA, USA), with intra- and interassay CVs of < 15% and < 18%, respectively. Free testosterone (FT) was calculated based on measured testosterone and SHBG serum concentrations using the equation described by Vermeulen et al. (1999), assuming a fixed albumin concentration of 43.8 g/L.

*Statistics*. In order to correct for urinary dilution, we adjusted BPA concentrations for the urinary osmolality, normalized to the median osmolality of all samples (0.86 Osm/kg). This was done for all samples with a measured BPA concentration above LOD using the following equation:

Osmolality adj. concentration_i_ (ng/mL_(osm)_) = (C_i_ × Osm_M_)/Osm_i_, [1]

where C_i_ is the excreted urinary BPA concentration in nanograms per milliliter (ng/mL) for the *i*th sample, Osm_M_ is the median osmolality of all samples, and Osm_i_ is the osmolality of sample *i*. Urinary BPA concentrations below LOD (*n* = 5) were not adjusted for osmolality, but substituted by LOD/_√_^–^2. Osmolality-adjusted BPA concentrations [nanograms per milliliter (osmolality-adjusted)], including the five samples below LOD, were divided into quartiles based on the distribution among the 308 men. Osmolality-adjusted BPA concentrations were also entered into the statistical model as a continuous variable and were, because of skewed distribution, log2-transformed to diminish the impact of outliers.

We calculated descriptive statistics for population characteristics by quartiles of BPA. Associations between BPA excretion and reproductive outcomes adjusted for potential confounders were estimated by means of multiple linear regression analysis. Results are presented with 95% confidence intervals (CIs).

To achieve variance homogeneity and normality in the distributions of the residuals, the reproductive hormones LH, E_2_, FSH, FT and the ratios T/E_2_, T/LH, FT/LH, and inhibin B/FSH were modeled as natural log-transformed dependent variables, and model coefficients were back-transformed to express the percentage change in these variables. Semen volume, sperm concentration, and total sperm count were cubic-root transformed and percentage morphologically normal spermatozoa were square-root transformed. T, inhibin B, SHBG, and percentage progressive motile spermatozoa were left untransformed because of an acceptable normal distribution in residuals.

We conducted tests for linear trend across quartiles in regression models for ordinal BPA quartiles using integer values (1–4). Bivariate analyses (chi-square and one-way analysis of variance) were used to identify potential confounding factors among the men in quartiles of urinary BPA concentrations. Covariates included in the final model were known from the literature to be important predictors of the outcomes in question [e.g., ejaculation abstinence time in analyses of sperm concentration and smoking; body mass index (BMI) for reproductive hormone analyses]. In addition, covariates determined by bivariate analyses to be related with either BPA concentration or at least one of the outcomes with a *p* < 0.20 were initially included and subsequently stepwise excluded if they did not change the estimate by > 10%. For consistency, we adjusted all models of associations between BPA and reproductive hormones for the same set of covariates: BMI (< 20.0, 20.0–24.99, ≥ 25), current smoking (yes: men smoking at least once a week/no: men smoking < once a week), and time of blood sampling (clock time, nearest hour).

The following potential confounders were included in the final models of urinary BPA concentrations in relation to semen parameters: varicocele (yes/no), self-reported genital conditions [torsion of the testes, epididymitis, or inguinal hernia (yes/no)], cryptorchidism (yes/no), and current smoking (yes/no). In addition, we also adjusted the models of semen volume, concentration, and total sperm count for ejaculation abstinence time (linear splines: piecewise linear intervals 0–48 hr, 48–96 hr and > 96 hr). We also adjusted the models of sperm motility for duration of time between ejaculation and analysis of the sample. Potential confounders examined, but not included in the final statistical model, were fever > 38°C within the last 3 months, caffeine intake (milligrams per day), age, history of sexually transmitted diseases, recent use of medication, ethnicity, maternal education, and maternal smoking during pregnancy.

BPA may leak from food packaging or beverage cans/bottles (Geens et al. 2010); therefore, we did not initially adjust for dietary factors and alcohol consumption because they may be a source of exposure. However, we repeated the multiple linear regression analyses adjusting for alcohol, cola, diet cola, other sodas, fast food (pizza, hamburger, and French fries) intake (modeled as shown in Supplemental Material, Table S1), as well as percentage energy intake from fat, protein, and carbohydrates. Furthermore, we also examined the confounding potential of urinary excretion of the phthalate metabolites monoethyl phthalate (MEP), monoisobutyl phthalate (MiBP), mono-*n*-butyl phthalate (MnBP), and monobenzyl phthalate (MBzP) as well as the sum of di(2-ethylhexyl) phthalate (DEHP) and diisononyl phthalate (DiNP) metabolites, by repeating the statistical analyses with adjustment for the phthalate metabolites as continuous variables.

The statistical model assumption of homogeneity of variance was examined graphically by means of residual plots, and the assumption of normality of the distribution of residuals was examined graphically by means of histograms and normal probability plots.

All data analysis was performed using SAS version 9.1 (SAS Institute Inc., Cary, NC, USA).

## Results

A total of 303 of 308 (98%) men had urinary BPA concentrations above the LOD. The median (5th–95th percentile) unadjusted BPA concentration was 3.25 ng/mL (0.59–14.89 ng/mL), and the median (5th–95th percentile) osmolality-adjusted concentration was 3.74 ng/mL_(osm)_ [0.85–14.61 ng/mL_(osm)_]. The highest osmolality-adjusted BPA concentration we observed was 78.81 ng/mL_(osm)_. The clinical and demographic characteristics were similar across the quartiles of osmolality-adjusted urinary BPA concentration (see Supplemental Material, Table S1).

**Figure 1:**
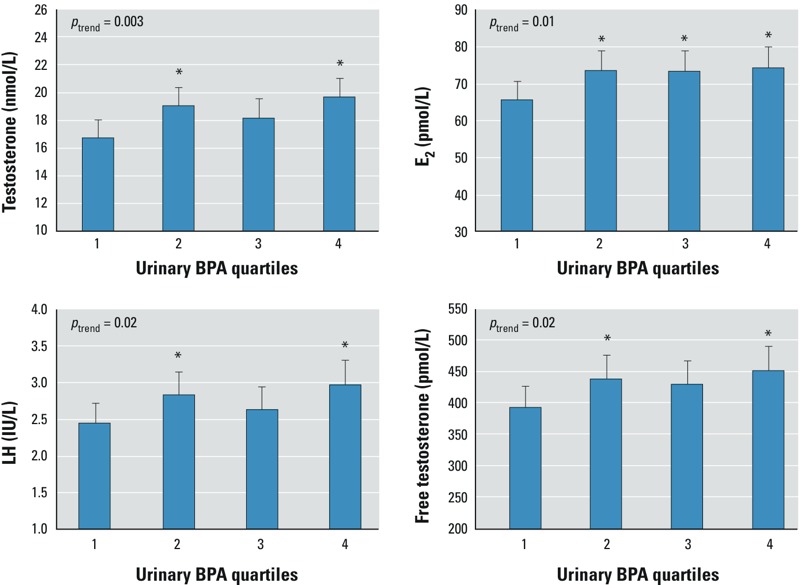
Selected reproductive hormones by quartiles of osmolality-adjusted urinary BPA concentration in 303 healthy, young men from the general population. Bars correspond to estimated median hormone values after adjusting for BMI, smoking, and time at day of blood sampling. Whiskers indicate 95% CIs. Range of BPA quartiles [ng/mL_(osm)_]: 1st, LOD–2.17; 2nd, 2.18–3.70; 3rd, 3.71–6.44; 4th, > 6.44. *p*_trend_, *p*-value for linear trend across quartiles of BPA. Note that the y-axes do not start at zero. *p < 0.05, compared with 1st BPA quartile.

*Associations between BPA and reproductive hormones*. Table 1 shows the associations between concentrations of urinary BPA and reproductive hormones. Men with urinary BPA concentrations above the lowest quartile had higher testosterone concentrations than men in the first BPA quartile. The differences in testosterone in nanomoles per liter between BPA quartiles presented in Table 1 correspond to 14% (95% CI: 4, 24%), 9% (95% CI: 1, 19%), and 18% (95% CI: 8, 28%) higher testosterone concentrations among men in the second, third, and fourth BPA quartiles, respectively, compared with testosterone concentrations of men in the lowest BPA quartile. Urinary BPA concentrations were also significantly positively associated with serum LH, E_2_, and FT concentrations (see also Figure 1). The positive associations between BPA and T, LH, E_2_ and FT were confirmed when modeling BPA as a continuous variable, although the estimate for LH did not reach statistical significance (Table 1). Serum FSH, inhibin B, SHBG, and ratios between hormones were not significantly associated with BPA exposure (Table 1; see also Supplemental Material, Table S2).

**Table 1:** Associations between reproductive hormones and osmolality-adjusted urinary BPA concentration in 303 healthy, young men from the general population (reported as untransformed model coefficients or percentage change with 95% CI and *p*-value).

BPA quartiles^*a*^	T (nmol/L)^*b*^	LH (IU/L)^*c*^	E_2_ (pmol/L)^*c*^	SHBG (nmol/L)^*b*^	FSH (IU/L)^*c*^	Inhibin B (pg/mL)^*b*^	FT (pmol/L)^*c*^
1st quartile	Reference	Reference	Reference	Reference	Reference	Reference	Reference
2nd quartile	2.3 (0.6, 4.0)	15.8% (1.2, 32.5%)	12.2% (2.5, 22.7%)	2.1 (–0.8, 5.0)	–3.0% (–18.1, 14.9%)	–9.3 (–28.4, 9.7)	11.4% (0.7, 23.3%)
*p*-Value	0.007	0.03	0.01	0.16	0.72	0.33	0.04
3rd quartile	1.5 (–0.2, 3.2)	7.7% (–5.9, 23.4%)	11.8% (2.1, 22.4%)	–0.4 (–3.3, 2.5)	–7.4% (–21.9, 9.7%)	–5.8 (–24.9, 13.3)	9.1% (–1.5, 20.7%)
*p*-Value	0.08	0.28	0.02	0.79	0.37	0.55	0.09
4th quartile	3.0 (1.3, 4.6)	21.6% (6.2, 39.1%)	13.4% (3.7, 24.1%)	2.1 (–0.8, 5.0)	12.7% (–4.8, 33.4%)	–10.6 (–29.6, 8.4)	14.8% (3.8, 27.0%)
*p*-Value	0.0006	0.005	0.006	0.16	0.16	0.27	0.008
*p*_trend_ across quartiles	0.003	0.02	0.01	0.42	0.25	0.35	0.02
Continuous^*d*^	0.7 (0.2, 1.1)	3.5% (–0.02, 7.1%)	2.7% (0.4, 5.1%)	0.6 (–0.1, 1.3)	2.6% (–1.7, 7.1%)	–3.8 (–8.6, 0.9)	2.7% (0.08, 5.3%)
*p*-Value	0.002	0.052	0.02	0.10	0.24	0.11	0.04
^***a***^Range of BPA quartiles [ng/mL_(osm)_]: 1st, LOD–2.17; 2nd, 2.18–3.70; 3rd, 3.71–6.44; 4th, > 6.44. ^***b***^Untransformed; adjusted for BMI, smoking, and time of day of blood sampling. ­^***c***^Transformed by the natural logarithm and back-transformed to obtain the percentage change. Adjusted for BMI, smoking, and time of day of blood sampling. ^***d***^The difference in hormone concentration associated with a doubling of the osmolality-­adjusted BPA concentration.							

**Figure 2:**
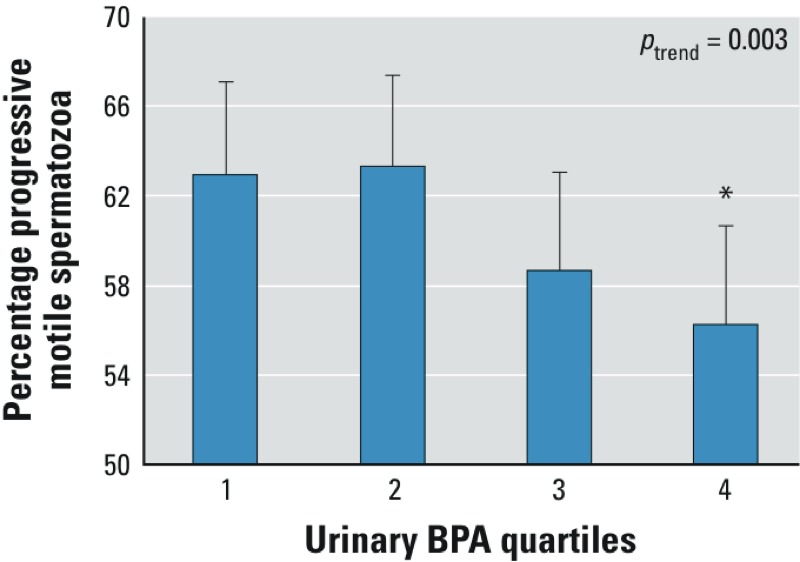
Percentage progressive motile spermatozoa by quartiles of osmolality-adjusted urinary BPA concentration in 293 healthy, young men from the general population. Bars correspond to estimated median percent progressive motile spermatozoa after adjusting for smoking, cryptorchidism, varicocele, genital conditions (torsion of the testes, epididymitis, or inguinal hernia) and time to motility analysis. Whiskers indicate 95% CIs. Range of BPA quartiles [ng/mL_(osm)_]: 1st, LOD–2.17; 2nd, 2.18–3.70; 3rd, 3.71–6.44; 4th, > 6.44. *p*_trend_: *p*-value for linear trend across quartiles of BPA. Note that the y-axis does not start at zero. *p < 0.05, compared with 1st BPA quartile.

*Associations between BPA and semen quality*. BPA excretion was not associated with semen volume, sperm concentration, total sperm count, or percentage morphologically normal forms (Table 2). However, we did observe a significant inverse association between BPA excretion and progressive motility (Table 2). Men in the fourth BPA quartile had significantly lower percentage progressive motile spermatozoa in their ejaculate compared with men in the first BPA quartile (–6.7 percentage points; 95% CI: –11.76, –1.63). This corresponded to a mean percentage progressive motile spermatozoa of 56% (95% CI: 52, 61%) versus 63% (95% CI: 59, 67%) in the highest versus the lowest BPA quartile groups after adjusting for smoking, genital conditions, and time to motility analysis (Figure 2). The trend test across quartiles of BPA was highly significant. The inverse association was confirmed when we modeled BPA concentration as a continuous variable (Table 2).

**Table 2:** Associations between semen quality parameters and osmolality-adjusted urinary BPA concentration in healthy, young men from the general population [reported as model coefficients (95% CI)].

BPA quartiles^*a*^	Semen volume (mL)^*b*^	Sperm concentration (million/mL)^*b*^	Total sperm count (million)^*b*^	Morphologically normal forms (%)^*c*^	Progressive motile (%)^*d*^
1st quartile	Reference	Reference	Reference	Reference	Reference
2nd quartile	–0.04 (–0.11, 0.03)	0.16 (–0.21, 0.52)	0.10 (–0.43, 0.69)	0.01 (–0.30, 0.32)	0.40 (–4.64, 5.43)
3rd quartile	0.00 (–0.07, 0.07)	–0.08 (–0.45, 0.29)	–0.07 (–0.60, 0.46)	–0.09 (–0.41, 0.22)	–4.25 (–9.31, 0.81)
4th quartile	–0.01 (–0.08, 0.06)	–0.04 (–0.41, 0.33)	–0.05 (–0.59, 0.48)	–0.01 (–0.33, 0.30)	–6.70 (–11.76, –1.63)*
*p*_trend_ across quartiles	0.95	0.56	0.71	0.79	0.003
Continuous^*e*^	0.00 (–0.02, 0.02)	–0.01 (–0.10, 0.09)	0.00 (–0.13, 0.14)	–0.02 (–0.10, 0.06)	–1.82 (–3.10, –0.53)*
^***a***^Range of BPA quartiles [ng/mL_(osm)_]: 1st, LOD–2.17; 2nd, 2.18–3.70; 3rd, 3.71–6.44; 4th, > 6.44. ^***b***^Cubic-root transformed; adjusted for smoking, varicocele, cryptorchidism, genital conditions, and ejaculation abstinence time. *n* = 297 (semen volume and total sperm count) and *n* = 298 (sperm concentration). ^***c***^Square-root transformed; adjusted for smoking, varicocele, cryptorchidism, and genital conditions. *n* = 296. ^***d***^Untransformed; model coefficients express the mean difference in percentage points between the percentage progressive motile spermatozoa in the given BPA quartile (2nd, 3rd, or 4th) and the reference group (1st quartile); adjusted for smoking, varicocele, cryptorchidism, genital conditions, and time to motility analysis. *n* = 293. ^***e***^The difference in semen quality associated with a doubling of osmolality-­adjusted BPA concentration. *p < 0.05.					

Results for all outcomes from unadjusted and adjusted models of associations were consistent (results not shown). Inclusion of alcohol; other dietary factors; or total energy intake, energy from fat, protein, or carbohydrates in the statistical models did not substantially change the associations between BPA and reproductive outcomes, although the association with FT was slightly attenuated when including alcohol [13.0% (95% CI: 2.4, 24.8%) higher FT in fourth vs. first BPA quartile after additional adjustment for alcohol]. We also repeated the analyses including phthalate metabolites as confounders, which also did not change the associations (data not shown).

## Discussion

We found that higher urinary BPA concentration was associated with significantly higher concentrations of serum LH, T, and E_2_ and a lower percentage of progressive motile sperm in healthy, young men.

It is well established by *in vitro* studies that BPA can interact with the estrogen receptors ERα and ERβ as a partial agonist and, in the presence of E_2_, exhibit competitive inhibition by blocking the binding of the more potent natural estrogen to the ERs (Gould et al. 1998; Li et al. 2012; Wetherill et al. 2007). If our observed positive associations between BPA and a concurrent increase in LH, T, and E_2_ are causal, they could be explained by BPA acting as an antiestrogen at the hypothalamic or pituitary level. A competitive inhibition of the binding of E_2_ to ERs in the hypothalamus/pituitary by BPA would lead to an attenuation of the negative feedback of circulating E_2_ on LH and FSH release, resulting in higher circulating LH concentrations, which would subsequently increase T (and E_2_) production by the testis. The lack of a concurrent statistically significant increase in FSH could be explained by the negative feedback of inhibin B on FSH release given that we observed no statistically significant association between BPA and inhibin B. However, among men in the highest exposed quartile there was a nonsignificant trend of increased FSH, possibly due to a concurrent trend of decreased inhibin B in the same quartile.

*In vitro* studies have also shown that BPA can act as an AR antagonist (Bonefeld-Jørgensen et al. 2007; Kitamura et al. 2005; Lee et al. 2003). A potential effect of BPA on the hypothalamic–pituitary–gonadal hormone axis could, therefore, also be mediated through an antagonistic effect of BPA on the AR, likewise resulting in the same hormonal pattern. For example, patients with partial androgen insensitivity will typically have increased LH, testosterone, and E_2_ serum concentrations (Amrhein et al. 1977; Hellmann et al. 2012). Alternatively, associations might be explained by a combination of antiestrogenic and antiandrogenic effects.

Evidence regarding the effects of adult BPA exposure on reproductive hormone concentrations in animal models is not conclusive. Some studies in rats have reported altered reproductive hormone concentrations, for example, increased plasma concentration of LH and reduced plasma concentration of testosterone (Qiu et al. 2013) or increased intratesticular testosterone (Tohei et al. 2001) after BPA exposure in adulthood, but a lack of changes in serum or testis testosterone or E_2_ concentrations has also been reported (Quignot et al. 2012). However, in addition to species differences, the exposure levels and exposure routes used in animal studies may differ from human exposure. Effects observed at higher exposure levels typical of many experimental studies may also differ from effects of lower levels of exposure consistent with environmental exposures in humans.

In support of our findings, Galloway et al. (2010) observed increasing serum T concentrations with increasing BPA concentrations measured in 24-hr urine samples among 307 Italian men 20–74 years of age. Galloway et al. (2010) also reported a nonsignificant (*p* = 0.075) positive association between BPA concentrations and FT. Likewise, Meeker et al. (2010a) reported positive trends (albeit nonsignificant) between BPA and serum T (*p* = 0.17) and serum LH (*p* = 0.07), respectively, among 167 male partners in subfertile couples. However, in contrast to our findings, Galloway et al. (2010) and Meeker et al. (2010a) observed no association between BPA and E_2_ concentrations. Further, Meeker et al. (2010a) found a significant positive association with FSH and an inverse association with inhibin B. We observed a similar pattern in the present study, but only among men in the highest exposed quartile, and the associations in the present study were not statistically significant (*p* = 0.16 for FSH and *p* = 0.27 for inhibin B). Mendiola et al. (2010) did not observe any association between BPA and FSH and inhibin B concentrations in a study among 302 fertile men. In those fertile men, Mendiola et al. (2010) observed a trend toward a positive association between BPA and LH in line with our findings, albeit in their study the trend was not statistically significant. In contrast to our findings, Mendiola et al. (2010) did not observe associations with T or E_2_ among the fertile men, whereas a significant inverse association between BPA and free androgen index (calculated as total T × 100/SHBG), presumably driven by a positive association between BPA and SHBG, was observed. Thus, although some consistency between studies exist (e.g., for the association between BPA and T), there are also discrepancies (e.g., the positive association between urinary BPA and serum E_2_ concentration observed in the present study was not observed in the other studies). However, in participants from a Korean biomonitoring program, significantly higher urinary estrogen concentrations were observed in a group of men with high urinary BPA concentrations (*n* = 50) compared with an age-matched group of men with low urinary BPA concentrations (*n* = 50) (Kim et al. 2013). The diverging results between studies may be due to differences in exposure levels; the median urinary BPA concentration in the present study was, for example, twice the concentration observed among the fertile men in the study by Mendiola et al. (2010). Study size and composition of the study populations (e.g., fertile vs. infertile men) may also lead to diverging results. Some of the observations may be chance findings, and more studies of comparable study populations are needed to see which associations remain consistent.

Our results support the possibility that human exposure to BPA at environmental levels might be sufficient to affect the endogenous hormone balance. Our study participants were adult men with a mature hypothalamic–pituitary–gonadal hormone feedback system in place, which is expected to be less vulnerable than an immature system (for example, in a prepubertal child), which may not be able to compensate for a potential BPA-mediated attenuation of endogenous estrogen signaling. In addition, BPA-mediated attenuation of endogenous estrogen signaling, if present, might vary between tissue types and depend on the distribution of ERα and ERβ in individual tissues. Li et al. (2012) showed *in vitro* that whereas BPA acted as a competitive inhibitor of E_2_ activity in cells transfected with human ERα, no inhibition of E_2_ activity by BPA was observed in cells transfected with human ERβ.

We found an inverse association between BPA and percentage progressive motile sperm, but we found no associations with other semen parameters including sperm number. To our knowledge, there are no previous studies on the possible association between BPA exposure and semen quality among men from the general population. However, in men recruited through an infertility clinic, Meeker et al. (2010b) also observed a suggestive declining trend in percentage progressive motile spermatozoa with increasing urinary BPA concentrations (*p* = 0.10). However, they also observed inverse associations between BPA exposure and sperm concentration and morphology (Meeker et al. 2010b). In contrast, Mendiola et al. (2010) found no associations between semen parameters and BPA exposure in fertile men (*n* = 375). In a study population consisting of Chinese men (*n* = 218) with and without occupational exposure to BPA, Li et al. (2011) reported inverse associations between BPA exposure and sperm concentration, total sperm count, sperm vitality, and progressive motile spermatozoa but no associations with semen volume or sperm morphology. Chen et al. (2013) found no association between urinary BPA concentration and idiopathic male infertility in a case–control study among Chinese men using fertile men as controls (*n* = 877 cases, *n* = 713 controls). Thus, the literature on associations between BPA and human semen quality shows heterogeneous results. As discussed for observations on reproductive hormones, this discrepancy between studies on semen quality may be due to differences in BPA exposure levels, study size, and, perhaps most importantly, composition of study population—especially with regard to selection according to fertility status. In addition, it is possible that associations only seen in a single study may be chance findings rather than true biological associations.

A number of studies in rodents have, in agreement with our findings, reported adverse effects of adult BPA exposure on sperm motility (Dobrzynska and Radzikowska 2013; Quignot et al. 2012). Adverse effects of BPA on other sperm parameters have also been reported (Al-Hiyasat et al. 2002; Dobrzynska and Radzikowska 2013; Qiu et al. 2013; Quignot et al. 2012).

Similar to our suggested biological explanation for the observed association between BPA and reproductive hormones, the observed association between urinary BPA and sperm motility might also be explained by an antagonistic effect on ERs. Estrogen and androgen activity play an important role in the regulation of water reabsorption in the efferent ducts and epididymis and thereby influence the composition of the seminal fluid (Hess et al. 2011), as also shown in ER knockout mice (Joseph et al. 2010) and after exposure to ER antagonists in primates (Shayu et al. 2005). Hypothetically, a disturbance in the estrogen- and androgen-dependent processes in the efferent ducts and epididymis through competitive binding by BPA could potentially impact the luminal milieu in the epididymis and adversely affect the maturation of spermatozoa and, thereby, the normal development of sperm motility.

We based our BPA exposure assessment on a single spot urine sample. Because of the rapid urinary excretion of BPA (Völkel et al. 2002, 2005), the urinary BPA measurements in the present study reflect the exposure status shortly before sampling of both urine and blood. Endogenous reproductive hormones are constantly regulated through negative feedback systems. Therefore, the urinary BPA measurements may reflect exposure in the relevant window for acute effects on hormone regulation, making it more likely to detect significant associations with hormonal outcomes. In contrast, studies with repeated individual measurements of urinary BPA excretion have shown that there is a relatively high individual variability in urinary BPA excretion over time (Lassen et al. 2013; Ye et al. 2011). The BPA concentration from a single spot urine sample may, therefore, not be a robust marker of individual average long-term BPA exposure. Because the duration of spermatogenesis is approximately 90 days, the exposure measurement used may fail to adequately reflect the exposure level during the relevant window for affecting, for example, sperm concentration. Thus, the lack of significant associations with this outcome may be due to the potential exposure misclassification.

Because a large number of analyses were performed in the present study, some of our observed associations could be chance findings due to multiple testing. However, our observed association of BPA with concurrent increased T, E_2_, and LH concentrations is a biological pattern that is consistent with a plausible mechanism through ER and/or AR antagonism.

The men in the present study were unaware of their BPA exposure, semen quality, and reproductive hormone concentrations, and the vast majority (92%) had no knowledge of their ability to achieve successful conception. Knowledge about exposure or outcomes is therefore unlikely to have influenced willingness to participate in the study.

Overall, men with high BPA concentrations did not differ markedly from the men with low BPA concentrations with respect to lifestyle factors or clinical characteristics, and adjustment for potential confounders changed the estimates very little. The primary source of BPA is through diet; therefore, the observed associations could potentially be confounded by dietary factors (Geens et al. 2012). However, adjusting for alcohol, other dietary factors, and energy intake from fat, protein, or carbohydrate did not substantially change the results.

Co-exposure from other environmental chemicals, if related to BPA exposure, could also influence the observed associations. From an earlier study (Joensen et al. 2012), we also had information on urinary phthalate metabolite excretion in the same men. In order to test whether the observed associations with BPA exposure was confounded by correlation between BPA and phthalate exposure, we conducted additional analyses where we adjusted for urinary phthalate metabolite excretion. This did not substantially change the findings, indicating that our results were not confounded by co-exposure to phthalates. We cannot, of course, exclude that BPA exposure could be related to yet unrecognized residual or unmeasured confounders.

## Conclusions

BPA exposure was associated with higher circulating concentrations of T, LH, E_2_, and FT as well as a lower percentage of progressive motile sperm in the present study population of young men. The concern for BPA exposure is generally related to its estrogenic properties. However, the pattern of associations observed with reproductive hormones in the present study suggests the possibility that at environmental BPA exposure levels an antiestrogenic or antiandrogenic effect, or both, of BPA on the hypothalamic–pituitary–gonadal hormone feedback system may be a potential mode of action, possibly through a competitive inhibition on the receptor level. A similar antiestrogenic mechanism of human BPA exposure levels in the epididymis is also a plausible biological explanation for the association with lower sperm motility. However, additional research is needed to confirm our findings and to further test the suggested potential mechanisms.

## Supplemental Material

(213 KB) PDF
