# Supplementary material for: Urinary Bisphenol A Levels in Young Men: Association with Reproductive Hormones and Semen Quality
Source: Environ Health Perspect. 2014 May 1;122(5):478–84. doi: 10.1289/ehp.1307309 (PMC4014766; doi:10.1289/ehp.1307309)
Supplement: (213 KB) PDF [file ehp.1307309.s001.pdf]

Environmental Health Perspectives doi:10.1289/ehp.1307309

Supplemental Material

Urinary Bisphenol A Levels in Young Men: Association with Reproductive Hormones and Semen Quality

Tina Harmer Lassen, Hanne Frederiksen, Tina Kold Jensen, Jørgen Holm Petersen, Ulla N. Joensen, Katharina M. Main, Niels E. Skakkebaek, Anders Juul, Niels Jørgensen, and Anna-Maria Andersson

Table of Contents Page

Table S1. Distribution of potential confounders according to quartiles of osmolality adjusted BPA concentration 2

Table S2. Ratios between reproductive hormones in relation to osmolality adjusted urinary BPA concentration in 303 healthy, young men from the general population 3

Table S1. Distribution of potential confounders according to quartiles of osmolality adjusted BPA concentration[a].

**Variables**	**Total: N (%) or mean ± SD**	**1st quartile: % or mean ± SD**	**2nd quartile: % or mean ± SD**	**3rd quartile: % or mean ± SD**	**4th quartile: % or mean ± SD**	**P[b]**
**Information obtained at physical examination**						
Period of abstinence < 48 hours	26 (8)	9	4	9	12	0.36
Varicocele grade 2 or 3	22 (7)	5	6	6	10	0.62
BMI (kg/m^2)						
< 20	41 (14)	15	13	19	8	
20 – 24.99	206 (68)	71	66	60	75	
≥ 25	56 (18)	15	21	21	17	0.41
**Information obtained from questionnaire**						
Fever > 38°C within the last 3 months	24 (8)	11	8	8	6	0.67
Age > 20 years	73 (24)	18	26	22	29	0.45
Alcohol intake > 21 units/week	95 (31)	26	29	34	35	0.57
Total caffeine intake > 300 mg/day	58 (18)	12	25	21	18	0.21
Maternal education level (years)						
< 9	13 (4)	1	8	4	4	
9-10	61 (20)	24	17	18	18	
> 10	204 (66)	65	64	71	65	
Missing	30 (10)	8	12	6	13	0.47
Current smoking	123 (40)	42	35	35	48	0.29
Exposure to mother's smoking *in utero*	85 (31)	33	30	21	41	0.09
Self-reported genital conditions[c]	22 (7)	5	7	11	7	0.64
Sexually transmitted diseases	44 (14)	18	16	14	9	0.41
Born with cryptorchidism	13 (4)	3	3	9	3	0.11

**Variables**	**Total: N (%) or mean ± SD**	**1st quartile: % or mean ± SD**	**2nd quartile: % or mean ± SD**	**3rd quartile: % or mean ± SD**	**4th quartile: % or mean ± SD**	**P[b]**
Dietary factors						
Cola intake > 1.5 L per week	56 (19)	15	17	21	22	0.64
Other sodas > 1.5 L per week	38 (13)	16	5	16	14	0.14
Weekly cola light intake	47 (16)	12	21	15	15	0.51
Weekly pizza intake	94 (31)	33	29	25	38	0.34
Weekly hamburger intake	59 (20)	25	19	14	21	0.39
Weekly french fries intake	52 (17)	19	19	13	18	0.75
Total energy intake (MJ)	9.4 ± 3.9	9.8 ± 5.0	9.3 ± 3.4	9.8 ± 5.6	8.9 ± 3.3	0.50
Total fat (% of energy)	31.4 ± 5.7	32.2 ± 5.6	31.5 ± 6.2	31.0 ± 5.6	30.7 ± 5.2	0.40
Saturated fat (% of energy)	13.3 ± 2.7	13.7 ± 2.7	13.6 ± 3.0	13.1 ± 2.6	13.0 ± 2.5	0.22
Protein (% of energy)	16.5 ± 3.1	16.1 ± 3.4	16.6 ± 3.0	17.1 ± 3.2	16.3 ± 2.9	0.24
Carbohydrate (% of energy)	56.0 ± 7.1	55.6 ± 7.2	55.8 ± 8.3	55.8 ± 6.4	57.0 ± 6.5	0.63

[a]Range of BPA quartiles ($ng/mL_{(osm)}$): 1st (LOD-2.17), 2nd (2.18-3.70), 3rd (3.71-6.44), 4th (> 6.44). N = 77 in each quartile. [b]p-value for differences in distribution (chi-square) or mean (one-way analysis of variance) of potential confounders by quartiles of BPA.[c]Torsion of the testes, epididymitis, or inguinal hernia.

Supplemental Material, Table S2. Ratios between reproductive hormones in relation to osmolality adjusted urinary BPA concentration in 303 healthy, young men from the general population (reported as percentage change with 95% CI).

**BPA quartiles**[a]	**T-E_2 ratio**[b]	**T-LH ratio**[b]	**Free T-LH ratio**[b]	**Inhibin B-FSH ratio**[b]
1st quartile	Reference	Reference	Reference	Reference
2nd quartile	1.1% (-7.1, 10.0%)	-2.1% (-14.6, 12.3%)	-3.8% (-16.8, 11.2%)	-3.2% (-24.8, 24.7%)
p-value	0.80	0.76	0.60	0.80
3rd quartile	-4.6% (-12.4, 3.8%)	-1.0% (-13.7, 13.5%)	1.2% (-12.5, 17.1%)	5.7% (-18.1, 36.3%)
p-value	0.27	0.88	0.87	0.67
4th quartile	2.8% (-5.5, 11.9%)	-4.1% (-16.3, 10.0%)	-5.6% (-18.3, 9.1%)	-14.5% (-33.6, 10.1%)
p-value	0.52	0.55	0.44	0.22
p-trend[c]	0.85	0.60	0.60	0.35
Continuous[d]	0.6% (-1.5, 2.8%)	-0.2% (-3.5, 3.3%)	-0.8% (-4.3, 2.9%)	-4.1% (-10.1, 2.2%)
p-value	0.58	0.93	0.68	0.19

[a]Range of BPA quartiles ($ng/mL_{(osm)}$): 1st (LOD-2.17), 2nd (2.18-3.70), 3rd (3.71-6.44), 4th (> 6.44). [b]Transformed by the natural logarithm and back-transformed to obtain the percentage change. Adjusted for BMI, smoking and time at day of blood sampling. [c]P-value for linear trend across quartiles of BPA. [d]The estimate represents the difference in the ratio between hormones associated with a doubling of the osmolality adjusted BPA concentration.
